# The impact of COVID-19 on firm risk and performance in MENA countries: Does national governance quality matter?

**DOI:** 10.1371/journal.pone.0281148

**Published:** 2023-02-06

**Authors:** Hamza Almustafa, Quang Khai Nguyen, Jia Liu, Van Cuong Dang

**Affiliations:** 1 The Department of Banking and Financial Sciences, Faculty of Economics and Administrative Sciences, The Hashemite University, Zarqa, Jordan; 2 School of Banking, University of Economics Ho Chi Minh City (UEH), Chi Minh City, Vietnam; 3 Faculty of Business and Law, Accounting and Financial Management, University of Portsmouth, Portsmouth, Hampshire, United Kingdom; 4 School of Public Finance, University of Economics Ho Chi Minh City (UEH), Chi Minh City, Vietnam; University of Naples L’Orientale, ITALY

## Abstract

This study investigated the impact of the COVID-19 crisis on firm risk and performance in different country-level governance qualities in the MENA region. Analyzing a sample of 739 non-financial listed firms in 12 MENA countries for the period 2011–2020, we found that the COVID-19 crisis negatively impacted the performance of firms, especially low-performance firms, in most industries, and increased firm risk in general. Moreover, we found that national governance quality plays an important role in mitigating the negative impact of the COVID-19 crisis on firm operations. Specifically, national governance quality reduces the negative impact of the COVID-19 crisis on firm performance and the positive impact of the crisis on firm risk. The results are consistent with our contention that national governance quality contributes to creating a positive environment for businesses activities and reducing economic shocks.

## 1. Introduction

The COVID-19 has had a serious impact on various dimensions, including healthcare, economy, employment, and transportation around the globe [1–3]. Recent literature on the effect of the COVID-19 pandemic on corporate output has arrived at the consensus that the pandemic has had devastating consequences on capital markets and the global economy. Narayan et al [2] showed that lockdowns, travel bans, and economic stimulus packages resulted in the stock markets of G7 countries taking a hit. Similarly, Shen et al [1] found that COVID-19 negatively impacted firm performance in China. Padhan and Prabheesh [4], through their literature survey on the economics of the pandemic, showed that COVID-19 has had adverse economic effects in general.

**Fig 1:**
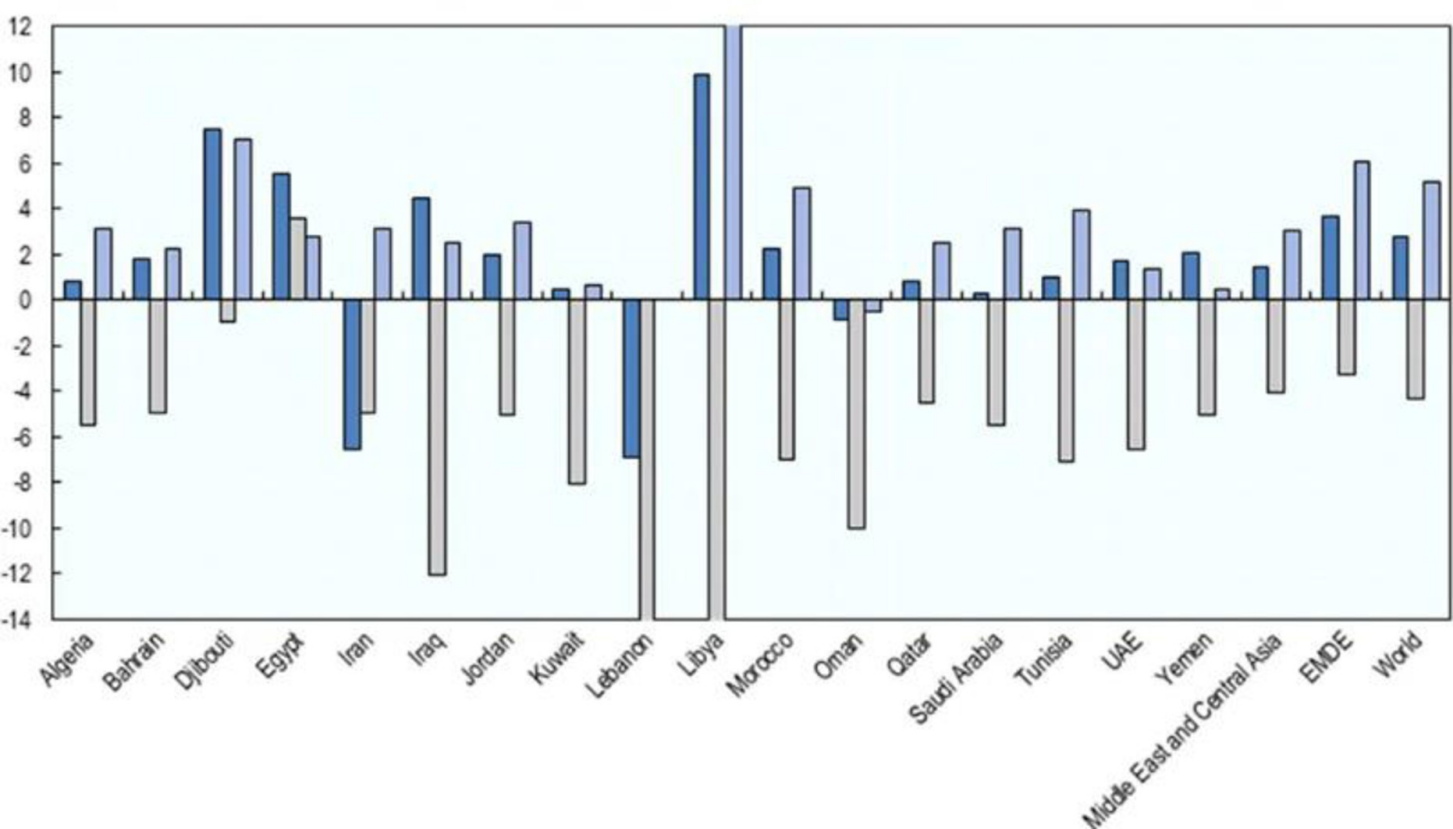
GDP in some MENA countries (year on year percent change). *Source: IMF, World Economic Outlook Database, OECD [8].* Note: 2019—Dark blue, 2020—Grey, 2021—Light blue.

**Fig 2:**
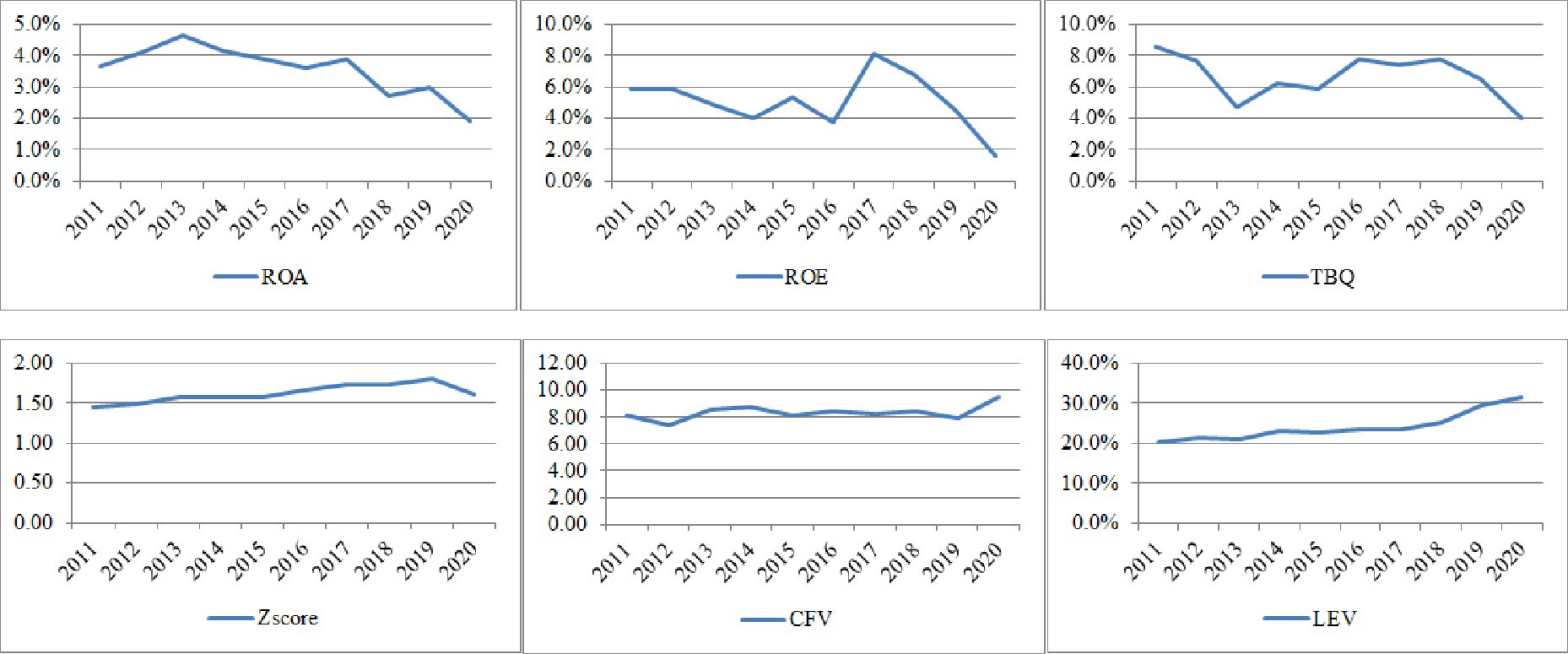
Firm risk and performance in MENA countries (average value). *Source*: *Authors’ calculation*.

Many studies have suggested that the COVID-19 crisis could have a more significant impact on the economy of Middle East and North Africa (MENA) countries than originally anticipated [5, 6]. Regarding the magnitude and speed of the outbreak across the MENA region, Usman et al [6] state that “the epidemic has serious consequences for the world economy and regional economies, particularly the oil-exporting states resulting in the downward revision of economic growth.” Yellinek [7] reports that MENA countries lost significant revenue from trade, tourism and hospitality, retail, expo, and real estate in 2020, causing long-term negative impact on these countries’ economies and destabilizing their financial institutions and businesses, among others. Some countries, such as Iran, Bahrain, Saudi Arabia, Oman, and UAE have also had multiple cases of infection, which have had a further impact on their economy. Based on World Economic Outlook Database, OECD [8] report that the gross domestic product (GDP) of most MENA countries has reduced significantly (Fig 1). Although the pandemic negatively impacted the economies of most countries, the degree of these effects is not the same. For example, Gao et al. [9] found that the impact of the COVID-19 crisis on financial markets differed between US and China because of the different epidemic management modes adopted in these countries. In addition, Fig 2 shows that firm performance, which is measured by ROA, ROE, and Tobin’s Q reduced significantly in 2020, and firm risk, which is measured by Z-score, CFV, and, LEV increased significantly indicating that COVID-19 crisis significant impact firms activities in MENA countries. While the impact of COVID-19 on the economies of MENA countries has been very severe and the impact of this crisis on business operations is complex, there is a lack of studies on the impact of COVID-19 on firm operations in this region.

By focusing on listed firms in MENA countries, this study contributes to the literature in three ways. First, although some studies have found that COVID-19 had a negative impact on firm performance in general [1, 10], this impact may differ from firm to firm. For example, Shen et al [1] found that the impact of COVID-19 on firm performance is more pronounced in firms with smaller sales revenues or investment scales. Using different estimation methods, including FE, System GMM, and quantile regression, our study contributes to the literature by conducting an in-depth investigation of the impact of the COVID-19 crisis on firm performance. Our results show that COVID-19 has an especially negative impact on firms with low levels of performance; however, there is a lack of clear evidence regarding the impact of COVID-19 on the performance of firms with high levels of performance.

Second, some studies have investigated risk during the COVID-19 crisis period, although most of these studies have focused on risk in the stock market [11, 12]. To the best of our knowledge, this is the first study to investigate the impact of the pandemic on firm risk. Using three firm risk measures, including the Z-score index, financial leverage, and cash flow volatility, as proxies of firm risk, we found that the COVID-19 crisis increased overall firm risk. This finding provides important implications for firms to enhance risk management effectiveness.

Finally, this is the first study to investigate whether the impact of the COVID-19 crisis on a firm’s operations differs from country to country, analyzed through the lens of the national governance system, and how national governance quality modifies the relationship between COVID risk and firm operations. Current literature shows that, apart from internal, firm-level characteristics and management activities, firm risk-taking activities can be influenced by the external, country-level environment [13]. Boubakri et al. [14] provided evidence that political institutions and connectedness influence corporate risk-taking. Ngobo and Fouda [15], Nguyen [16] and Nguyen and Dang [17] have shown that improvements in a country’s institutional quality positively affect corporate performance and profitability and reduce risk. Our study extends this stance of the literature to examine how country-level governance influences the relationship between COVID-19, firm risk, and firm performance in a region where the national governance quality is diversified.

The remainder of this paper is organized as follows. Section ‎2 presents the literature review and develops the research hypotheses. Section 3 discusses the data and sample and develops the research models. The results and discussion are presented in Section ‎4. Finally, Section 5 concludes the paper and provides research implications.

## 2. Literature review and hypothesis development

### 2.1 COVID-19 and firm performance

The COVID-19 pandemic is a major health emergency and has resulted in worldwide economic crises. From a management perspective, the behavioral theory of the firm implies that managers might face cognitive limitations during an economic crisis. Economic crises with higher levels of uncertainty may reduce the information processing abilities of managers and lead them to make bad decisions [18]. Many countries introduced pandemic control policies that affected the production and business activities of firms around the world. Specifically, people across countries were asked to go out less, and mass production and business activities were prohibited to prevent viral transmission. These policies had a significant negative impact on aggregate demand, especially export and consumption [1].

Studies have provided evidence that firm performance in some countries reduced during the crisis. Shen et al [1] found that the pandemic had a negative impact on Chinese firm performance, and Ren et al [10] provided evidence that the Chinese equity market plummeted and was roiled in crisis due to low expected firm performance and the rapid spread of COVID-19. However, they agree that although strict lockdown restrictions led to deteriorating economic prosperity in China, negative effects on firm values are temporary. Gu et al. [19] found that the negative impact of the COVID-19 crisis on corporate performance is especially severe in some industries, such as software, construction, computer services, and information transfer. Although firms in some industries, such as healthcare and e-commerce, may be positively impacted by COVID-19, we expect the pandemic to have negatively affected firm performance in general. Thus, we propose the following hypothesis:

*H1: COVID-19 is negatively associated with firm performance*.

### 2.2 COVID-19 and firm risk

The COVID-19 crisis created an economic shock that diminished expected and actual costs, sales, and profits, putting downward pressure on the value of firms’ assets and increasing operational risk. Many previous studies based on the behavioral theory of the firm and prospect theory argue that firms may respond to economic adversity by undertaking greater, not lower, risk [18, 20]. Besides, the injection of liquidity provided in loan guarantees and new lines of credit, which are applied in many countries, increased firms’ leverage ratios as well as their default risk [21] also imply that the economic shock resulting from the COVID-19 crisis is likely to translate into an enduring risk of a wave of corporate insolvencies as well as a significant increase in leverage. Some empirical studies provide evidence that the COVID-19 crisis may increase firm risk. Banerjee et al. [22] find that the pandemic has placed enormous strains on corporate cash buffers: 50% of the firms in their sample did not have sufficient cash to cover total debt servicing costs over the upcoming year. Moreover, sticky operating expenses during the COVID-19 crisis period resulted in many firms incurring operating losses, placing an additional burden on cash buffers and increasing liquidity risk. Based on these arguments, we expect that the COVID-19 crisis increased firm risk in general; thus, we propose the second hypothesis, as follows:

*H2: COVID-19 is positively associated with firm risk*.

### 2.3 National governance quality, firm risk, and performance

Following the institutional quality theory, previous studies proposed that national governance quality can enhance the efficiency of firm management [17, 23]. LiPuma et al. [24] provided evidence that institutional quality or national governance quality facilitate export performance. SN and Sen [25] found that corruption negatively influences firm productivity in India. High national governance quality engenders a good environment for firms to engage in business activities. Williams [26] investigated the relationship between national governance and banks’ risk-taking behavior in Asia and found that national governance is negatively related to banks’ risk-taking. Tran [13] examined the relationship between corruption and corporate risk-taking using data from 20 emerging markets and found that corruption negatively affects firm-level risk-taking activities. Boubakri et al [14] showed that weak political institutions are associated with risk-taking activities worldwide. These findings support the contention that strong political institutions reduce government rent-seeking, the resulting outright expropriation of corporate resources, and external financing costs. The impact of the COVID-19 crisis differed with the institutional environment [27]. Hence, we hypothesize that national governance is associated with the impact of the COVID-19 crisis on firm risk and performance, as follows:

*H3a: National governance quality is negatively associated with the effects of the COVID-19 crisis on firm performance*.
*H3b: National governance quality is negatively associated with the effects of the COVID-19 crisis on firm risk*.

## 3. Methodology

### 3.1 Sample and data

We obtained the financial accounts data from Thomson Reuters Eikon, “Our World in Data”, and the national governance data from World Bank’s Worldwide Governance Indicators (WGI). Our sample consisted of 739 listed, non-financial firms in 11 MENA countries, namely, Bahrain, Egypt, Jordan, Kuwait, Morocco, Oman, Qatar, Saudi Arabia, Tunisia, Turkey, and United Arab Emirates, for the period 2011–2020, with a total of 3253 firm-year observations. We excluded financial firms, firms with missing data, and firms with missing data during the 2019–2020 period. The firms in our data were classified into 17 industries (Appendix 2 in S1 Appendix). Most listed firms in these countries are usually large, multinational, or have a wide range of businesses in the MENA region. Therefore, using the data of listed firms will help better assess the impact of COVID-19 on firm operations.

### 3.2 Variables measures

#### 3.2.1 Firm performance measures

We employed both market-based and accounting-based performance measures, including Tobin’s Q (TBQ), return on equity (ROE), and return on assets (ROA). TBQ is measured as the ratio of the sum of the market value of equity and the book value of total liabilities to the total book value of both equity and liabilities. TBQ proxies market expectations about the firm’s future earnings; a high TBQ value implies that investors have high expectations about the company’s future prospects. Thus, it is known as a market-based measure of firm performance. ROA and ROE are computed as the ratio of profit before tax to total assets and total equity, respectively. Higher values of ROA and ROE, which are known as accounting-based measures of firm performance, imply that firms effectively use capital and assets to generate profits; thus, the higher the ROA and ROE, the higher the firm performance. These measures are widely used in the literature [28–32].

#### 3.2.2. Firm risk measures

We employed three measures for firm risk. First, following Harris and Roark [33], Bates et al. [34], Harris and Roark [33], and Ozkan and Ozkan [35], we used cash flow volatility (CFV), which is calculated as the standard deviation of cash flow to assets ratio for each firm over the sample period. A higher value of CFV implies that the firm’s cash flow is not stable, and thus, the firm has higher risk. Second, we followed Díez-Esteban et al. [36] and used the Z-score as an alternative measure of firm risk, which is calculated as the sum of the return on assets ratio and the capital asset ratio divided by the standard deviation of the return on assets ratio over the entire sample period. A higher Z-score value indicates that firms are more stable and less risky[37, 38]. Finally, we used leverage (LEV), calculated as the ratio of total debt to total assets, to measure financial risk. A higher LEV value indicates higher level of firm risk [39, 40].

#### 3.2.3 COVID-19 crisis measures

To investigate the impact of the Covid-19 crisis on firm risk and performance, we use a dummy variable as the independent variable, which is 1 for the Covid-19 period or 0 otherwise. Shen et al [1] states that the impact of the Covid-19 crisis on the economy is clear in 2020, although the Covid-19 outbreak started in 2019. In the MENA region, the countries have taken steps to control the pandemic at the beginning of 2020. We, therefore, determine the Covid-19 crisis period is 2020, as Shen et al [1] suggested. In addition, we also use the stringency index (SINDEX) to measure the severity of COVID-19 crisis as robustness test. We applied the natural logarithm to scale this variable.

#### 3.2.4 National governance quality measures

We adopted the World Governance Index (WGI) provided by World Bank to measure national governance quality. WGI is a value-weighted average of six components of country-level governance quality, which include voice and accountability, political stability and absence of violence/terrorism, government effectiveness, regulatory quality, the rule of law, and control of corruption. These indicators are displayed in standard normal units ranging from 2.5% to +2.5, with a larger value indicating better national governance quality. However, research argues that these indicators are highly correlated [41, 42], thus making it difficult to include them in one regression, and using the average indicates may lead to bias in the results. Therefore, following Hou and Wang [43], we used each of single components, i.e., voice and accountability (VA), political stability and absence of violence/terrorism (PV), government effectiveness (GE), regulatory quality (RQ), the rule of law (RL), and control of corruption (CC)., to proxy national governance quality as a robustness test.

#### 3.2.5 Control variables

We utilized firm age and firm size in our empirical models to control the firm’s “scope of operation” that may affect firm management activities [16, 44, 45] and firm risk and performance. Firm age is defined as the total number of years in a corporation, whereas firm size is defined as the natural logarithm of a firm’s market capitalization. Second, we controlled for investment opportunities using the market-to-book assets ratio (MB) value. MB is defined as the sum of equity market value and debt book value divided by the sum of the book values of equity and debt [36]. Investment opportunities are found to affect firm risk [46–48].

We also account for dividends by including a dummy variable equal to 1 for a dividend-paying company and 0 otherwise. Previous studies have agreed that dividend policy is associated with firm risk and performance [49–51]. Furthermore, prior studies have found that asset structure can affect firm risk and performance [52, 53]; therefore, we controlled it using the tangible assets ratio, defined as the ratio of tangible fixed assets to total assets. To control the debt structure that may affect firm risk and performance [54, 55], we used the ratio of long-term debt to total debt. Furthermore, in regression models used to investigate the impact of the COVID-19 crisis on firm performance, we also controlled capital structure by using the firm’s financial leverage ratio as a control variable, as firm leverage was found to be an important factor affecting firm performance [56–59]. Finally, since the levels of economic development of countries in the MENA region are very different, the impact of the COVID-19 crisis on the countries’ economics may also be different. We, therefore, use the logarithm of GDP per capita to control the level of economic development in our models. The definitions of all variables are presented in Table 1.

**Table 1:** Variable definitions.

Variable	Definition
Firm risk	
Z-score index (Z-score)	The sum of the return on assets ratio and the capital asset ratio divided by the standard deviation of each firm’s return on assets ratio over the entire sample period.
Cash flow volatility (CFV)	The standard deviation of cash flow to asset ratio of each firm over the entire sample period.
Firm leverage (LEV)	The total debt scaled by total assets.
Firm performance	
Return on assets (ROA)	The ratio of profit before tax to total assets.
Return on equity (ROE)	The ratio of profit before tax to total equity.
Tobin’s Q (TBQ)	The ratio of the sum of the market value of equity and the book value of total liabilities to the total book value of both equity and liabilities.
COVID-19 crisis	
Covid-19 occurrence (COVID)	The dummy variable of “outbreak time” is 1 for 2020, and 0 otherwise.
Stringency index (SINDEX)	The natural logarithm of the stringency index which is a composite measure based on nine response indicators including school closures,workplace closures, and travel bans, rescaled to a value from 0 to 100. This index was collected from “Our World in Data”
National governance quality	
Overall index (WGI)	The composite score includes the six governance dimensions: voice and accountability (VA), political stability and absence of violence/terrorism (PV), government effectiveness (GE), regulatory quality (RQ), the rule of law (RL), and control of corruption (CC).
Control variables	
Firm age (FAGE)	The total number of years in corporations.
Market-to-Book ratio (MTB)	The sum of the equity market value and the debt book value divided by the sum of the book values of equity and debt.
Firm size (FSIZE)	The natural logarithms of the firm’s market capitalization.
Dividends (DIV)	Dummy variable equals 1 for dividends-paying company, and 0 otherwise.
Tangibility (TANG)	The ratio of tangible fixed assets to total assets.
Debt structure (DEBT)	The ratio of long-term debt to total debt.
Industry group (INDS)	Dummy variable equals 1 if the industry of the firm is “Food,” “Health Care Equipment & Services,” “Pharmaceuticals, Biotechnology & Life Sciences,” “Software & Services,” or “Telecommunication Services,” and 0 otherwise.
Economic development (GDPC)	The natural logarithm of GDP per capita.

### 3.3 Empirical model and estimation methods

To analyze the impact of the COVID-19 crisis on firm risk and performance, we tested our three hypotheses using the following regressions:

$${{PERF}_{i,t}=\alpha }_{0}+\alpha_{1}{COVID}_{j,t}+\alpha_{j}C_{i,t}+\mu_{c}+\theta_{k}{+\epsilon }_{i,t}$$ (1)

$${{FRISK}_{i,t}=\beta }_{0}+\beta_{1}{COVID}_{j,t}+\beta_{j}C_{i,t}+\mu_{c}+\theta_{k}+\epsilon_{i,t}$$ (2)

Where PERF and FRISK are dependent variables firm performance and risk, respectively; COVID is the COVID-19 crisis; C is a vector of control variables; *α*_0_ and *β*_0_ are constant; *α*_j_ and *β*_j_ are unknown estimated coefficients; μ_*c*_ represents unobserved countries fixed-effect; θ_*k*_ represents industry fixed-effect; and *ϵ*_*i*,*t*_ represents the independent error term. All variables are defined in Section 3.2 and summarized in Table 1.

Further, to analyze the role of national governance quality in moderating the impact of the COVID-19 crisis on firm risk and performance, we introduced an interaction term of COVID-19 and national governance quality, COVID*WGI, to test Hypothesis 3 by using the following regressions:

$${{PERF}_{i,t}=\gamma }_{0}+\gamma_{1}{COVID}_{j,t}+{\gamma_{2}WGI+\gamma_{3}COVID*WGI+\gamma }_{j}C_{i,t}+\mu_{c}+\theta_{k}+\epsilon_{i,t}$$ (3)

$${{FRISK}_{i,t}=\delta }_{0}+\delta_{1}{COVID}_{j,t}+{\delta_{2}WGI+\delta_{3}COVID*WGI+\delta }_{j}C_{i,t}+\mu_{c}+\theta_{k}+\epsilon_{i,t}$$ (4)

Where PERF and FRISK are dependent variables firm performance and risk, respectively, WGI represents national governance quality; C is a vector of control variables; *γ*_j_ and *δ*_j_ are unknown estimated coefficients; μ_*c*_ represents unobserved countries fixed-effect; θ_*k*_ represents industry fixed-effect; and *ϵ*_*i*,*t*_ represents the independent error term.

We performed Hausman tests, which suggested that the fixed effect model is appropriate for our estimation model. Therefore, we applied the fixed effect (FE) for models (1), (2), and (3). The FE model controls individual effects and enabled us to capture individual heterogeneity [57]. We ran these models with the specification of robust standard errors for country clusters.

As robustness tests, first, to treat the potential endogeneity in our data, we applied the system GMM regression and performed specification and validation tests to ensure its compatibility and efficiency in our empirical analyses. The two-step system GMM technique involves a system of equations in differences and levels that allowed us to treat all the explanatory variables under categories. Second, we applied the quantile regression method to investigate the heterogeneous effects of COVID-19 on firm risk and performance and the role of country governance quality.

## 4. Results and discussion

### 4.1 Descriptive statistics and correlation matrix

Table 2 illustrates the descriptive analysis of the variables used in our investigation. The basic statistics show that firms, on average, have a relatively low ROA mean (0.05) across the sampling period and a 0.16 average ROE. However, the firms in our sample seem to have a relatively high average Tobin’s Q ratio (9.23), suggesting that firms add value to their shareholders across the selected emerging markets. The descriptive analysis also illustrates that the average Z-score and cash flow volatility CFV are 3.60 and 9.15, respectively, while the mean financial leverage and debt structure are 0.29 and 0.33, with a maximum 0.80 and 0.72, respectively. Finally, the descriptive statistics of the national governance index suggest that firms in our sample belong to different institutional quality systems. The average value of the national governance index is 0.10, with a minimum of -1.72 and a maximum of 1.48, suggesting the institutional quality background diversity across our sample. In addition, firms have 32 years on average age and 3.19 of growth opportunities, 8.31 for firm size, and 1.25 for tangibility.

**Table 2:** Descriptive statistics.

Variable	Obs	Mean	Std. Dev.	Min	Max
ROA	3,253	0.048	0.123	-1.641	0.210
ROE	3,253	0.160	2.333	-0.580	0.470
TBQ	3,253	9.230	18.970	1.160	65.034
Z-score	3,253	3.601	13.886	0.000	25.684
LEV	3,253	0.294	0.237	0.018	0.800
CFV	3,253	9.149	13.523	0.023	16.801
COVID	3,253	0.193	0.395	0.000	1.000
SINDEX	3,253	0.559	3.347	0.000	4.062
WGI	3,253	0.097	0.683	-1.717	1.481
FAGE	3,253	32.270	21.938	8.000	120.000
MTB	3,253	3.191	14.861	0.000	36.333
FSIZE	3,253	8.306	0.761	5.386	10.957
DIV	3,253	0.642	0.480	0.000	1.000
TANG	3,253	1.247	16.343	0.000	0.420
DEBT	3,253	0.335	2.334	0.012	0.720
INDS	3,253	0.273	0.445	0.000	1.000
GDPC	3,253	9.234	0.904	7.802	11.351

Note: This table reports descriptive statistics of the main variables used in the empirical models—Obs, mean, Std. Dev, min, and max are the number of observations, mean value, standard deviation, mean value, and max value of the variable, respectively. All variables are defined in Table 1.

The correlation matrix reported in Table 3 provides preliminary evidence on the impact of COVID-19 on firm risk and performance and the effect of other explanatory variables. As reported in Table 2, all independent variables are statistically significantly correlated with the dependent variable, supporting the proposition that these independent variables are important determinants of a firm’s risk and its performance. COVID-19 has a strong correlation with the firm’s performance, measured by ROA on the one hand, and with the firm’s risk, measured by Z-score and corporate cash flow volatility (CFV) on the other hand. Importantly, the correlation analysis reveals a significant correlation between national governance (overall index) and our variables of interest, including COVID-19. The correlation coefficients among the independent variables revealed in Table 3 suggest that multicollinearity is not a serious problem in our empirical models as none of these coefficients exceeds the 0.80 threshold. Therefore, there is no considerable collinearity among the governance variables included in our model.

**Table 3:** Correlation matrix.

Variables	ROA	ROE	TBQ	Z-score	LEV	CFV	COVID	SINDEX	WGI	FAGE	MTB	FSIZE	DIV	TANG	DEBT	GDPC
ROA	1.000															
ROE	0.050	1.000														
	(0.000)															
TBQ	-0.024	-0.001	1.000													
	(0.018)	(0.894)														
Z-score	-0.018	-0.006	0.009	1.000												
	(0.086)	(0.539)	(0.378)													
LEV	-0.100	-0.018	-0.003	0.182	1.000											
	(0.000)	(0.172)	(0.820)	(0.000)												
CFV	0.001	0.002	0.128	-0.055	0.072	1.000										
	(0.923)	(0.848)	(0.000)	(0.000)	(0.000)											
COVID	-0.055	0.003	0.005	0.142	0.100	0.000	1.000									
	(0.000)	(0.790)	(0.616)	(0.000)	(0.000)	(1.000)										
SINDEX	0.009	0.003	0.000	0.045	0.012	0.001	0.335	1.000								
	(0.376)	(0.780)	(0.986)	(0.000)	(0.351)	(0.948)	(0.000)									
WGI	0.029	0.003	-0.009	0.012	0.039	0.030	0.030	0.020	1.000							
	(0.005)	(0.793)	(0.390)	(0.260)	(0.003)	(0.004)	(0.004)	(0.050)								
FAGE	0.017	0.012	0.001	0.047	-0.001	-0.032	0.081	0.030	0.100	1.000						
	(0.097)	(0.246)	(0.923)	(0.000)	(0.932)	(0.002)	(0.000)	(0.004)	(0.000)							
MTB	-0.105	-0.017	0.008	0.026	0.053	-0.006	0.020	0.003	-0.010	0.002	1.000					
	(0.000)	(0.098)	(0.420)	(0.011)	(0.000)	(0.540)	(0.052)	(0.775)	(0.322)	(0.874)						
FSIZE	0.121	0.005	-0.023	0.027	0.164	0.119	-0.002	0.001	0.327	-0.003	-0.054	1.000				
	(0.000)	(0.663)	(0.029)	(0.011)	(0.000)	(0.000)	(0.825)	(0.931)	(0.000)	(0.815)	(0.000)					
DIV	0.282	0.026	-0.019	0.071	0.079	0.047	-0.001	0.000	0.048	0.043	-0.050	0.293	1.000			
	(0.000)	(0.010)	(0.066)	(0.000)	(0.000)	(0.000)	(0.922)	(0.989)	(0.000)	(0.000)	(0.000)	(0.000)				
TANG	-0.003	0.001	0.538	-0.028	-0.012	0.335	-0.012	-0.001	0.011	-0.016	-0.003	0.039	0.011	1.000		
	(0.805)	(0.948)	(0.000)	(0.029)	(0.493)	(0.000)	(0.374)	(0.934)	(0.395)	(0.211)	(0.825)	(0.002)	(0.377)			
DEBT	-0.058	-0.018	0.019	0.178	0.426	0.045	0.139	0.036	0.091	-0.031	-0.004	0.363	0.109	-0.021	1.000	
	(0.000)	(0.186)	(0.145)	(0.000)	(0.000)	(0.001)	(0.000)	(0.007)	(0.000)	(0.019)	(0.740)	(0.000)	(0.000)	(0.221)		
GDPC	0.007	0.015	-0.034	-0.017	-0.031	0.013	-0.051	-0.029	0.327	-0.132	-0.025	0.355	-0.007	0.007	0.062	1.000
	(0.478)	(0.151)	(0.001)	(0.090)	(0.019)	(0.207)	(0.000)	(0.004)	(0.000)	(0.000)	(0.014)	(0.000)	(0.508)	(0.582)	(0.000)	

### 4.2 The impact of COVID-19 on firm risk and performance

#### 4.2.1 COVID-19 and firm performance

Table 4 presents the estimation results of Eq 1. The results show that the coefficient of COVID is negative with ROA, ROE, and TBQ but only statistically significant with ROA, suggesting that the COVID-19 crisis negatively affects firm performance, although there is no strong evidence to this end. Therefore, the first hypothesis (H1) was weakly supported. The coefficient of COVID in regression 1 is -0.002 indicating that Covid-19 crisis decreases the ROA by 0.2% on average. This effect seems to be not much in the short term. The occurrence of Covid-19 can cause heterogeneous damage to different industries. In particular, during this period, firms in MENA countries can be supported by government orders to reduce the impact of the crisis. Therefore, the negative impact of Covid-19 on firm performance is not immediate. In general, the results of our research show that the impact of COVID-19 on firm performance is not clear in the short term. As per the March 2021 World Bank report, most firms in MENA countries (80–90%) seem to have reopened in some capacity after a period of lockdown. However, World Bank also reported that the revenue of firms in this region declined significantly. Therefore, it is possible that the impact of COVID-19 on firm performance is heterogeneous across firms.

**Table 4:** COVID-19 and firm performance results.

	ROA		ROE		TBQ	
	(1)		(2)		(3)	
	Coef.	t-stat	Coef.	t-stat	Coef.	t-stat
COVID	-0.002**	-2.030	-0.079	-0.010	0.086	0.010
FAGE	0.073***	4.590	-2.378	-1.150	-3.393**	-2.110
MTB	-0.005	-0.630	-0.022	-0.020	-0.052	-0.040
FSIZE	0.001***	-4.870	0.003***	2.540	0.001**	1.910
DIV	0.058***	3.720	3.031	1.500	-5.518*	-1.840
TANG	0.016***	2.620	-0.031	-0.040	-1.354	-1.150
DEBT	-0.000	-0.340	-0.001***	-2.100	-1.823***	-4.740
LEV	-0.004	-0.510	0.202	0.180	5.424***	3.200
WGI	0.025***	2.420	0.070	0.050	0.667**	1.940
GDPC	-0.026***	-2.370	0.505	0.200	1.681	0.450
Cons	-0.050	-0.150	-28.828	-0.680	33.265	0.530
Country fixed effect	yes		yes		yes	
Industry fixed effect	yes		yes		yes	
Obs	3253		3253		3253	

Note: This table presents the results of the fixed effect estimates of Eq 1. See Table 1 for variable definitions. Superscripts *, **, *** indicate statistical significance at 10%, 5%, and 1% levels, respectively.

Regarding control variables, we find that the coefficient on FSIZE is positive and significant with all firm performance measures. The value of these coefficients is from 0.001 to 0.003 indicating that firm size can increase firm performance but not much. This finding is consistent with previous studies [60, 61] that large firms have the advantage of improving operational efficiency and increasing firm performance. The coefficients on DEBT are negative and statistically significant on ROE and ROA. Especially, the value of coefficient of DEBT in regression 3 is -1.823 indicating that long term debt reduces Tobin-Q significantly. Furthermore, the coefficient of WGI is positive and significant with firm performance measured by ROA and Tobin’s Q, indicating that country governance quality positively affects firm performance. Moreover, the value of these coefficients are quite high (0.025 and 0.006 in regressions 1 and 3, respectively) indicating that the effect of national governance quality on firm performance is significant. This result supports the institutional quality theory that firms operate better in a higher institutional quality environment.

#### 4.2.2 COVID-19 and firm risk

Table 5 presents the estimating results of Eq 2 using the FE method. The main results show that the coefficients on COVID are negative with Z-score but positive with LEV and CFV. All coefficients are statistically significant, indicating that the COVID-19 crisis increased overall firm risk. The value of these coefficients is quite high (-0.882, 0.107 and 0.001 in regressions 1, 2, and 3, respectively) indicating that Covid-19 significantly increases firm risk. Specifically, COVID-19 crisis pandemic significantly reduces firm stability as well as makes firms borrow more. The fact that Covid-19 has disrupted production activities in MENA countries and made it difficult for firms to pay due debts [62], thereby forcing firms to borrow more. Overall, these results strongly support hypothesis H2 and are consistent with the expectations of previous studies [21, 22]. As per our expectation, COVID-19 negatively affects the entire operation of businesses in MENA countries, thereby increasing risk. The revenue of firms reduced significantly during the COVID-19 crisis period. Several firms in MENA countries had to close down due to lockdowns, making it difficult for them to pay their debts as well as increase loans due to a serious lack of capital [63]. Moreover, the COVID-19 crisis led to banks in MENA countries increasing capital ratio [64], making it further difficult for firms in this region to access bank capital, leading to increased risks. Our research results provided strong evidence that the impact of COVID-19 on the economy was reflected not just in specific numbers, such as revenue and corporate profits, but also that the crisis increased the risk of firms that need to focus on avoiding system collapse. This empirical finding provides important implications for firms and regulators, primarily that firms need to focus on risk management, and regulators need to have appropriate policies to support firms to prevent systemic collapse in the aftermath of economic shocks.

**Table 5:** COVID-19 and firm risk results.

	Z-score		LEV		CFV	
	(1)		(2)		(3)	
	Coef.	t-stat	Coef.	t-stat	Coef.	t-stat
COVID	-0.882**	-2.080	0.107***	2.880	0.001***	4.150
FAGE	0.178	1.840	0.016***	2.110	0.000***	3.840
MTB	-0.000	-0.180	0.000***	2.690	0.012	0.330
FSIZE	-0.250**	-1.930	0.150***	5.020	0.001**	2.280
DIV	0.070	0.880	-0.017	-1.450	-0.001	-1.590
TANG	-0.000	-0.030	0.001	1.000	-0.024	-0.020
DEBT	-0.044	-0.390	0.185***	11.100	0.001**	1.950
WGI	-0.369*	-1.770	0.118***	3.850	-0.001	-0.930
GDPC	-0.077	-0.300	0.063	1.700	0.001***	8.580
Cons	-2.269	-0.530	-2.053***	-3.230	9.156***	14.000
Country fixed effect	yes		yes		yes	
Industry fixed effect	yes		yes		yes	
Obs	3253		3253		3253	

Note: This table presents the results of the fixed effect estimates of Eq 2. See Table 1 for variable definitions. Superscripts *, **, *** indicate statistical significance at 10%, 5%, and 1% levels, respectively.

Table 5 also shows that firm size is positively associated with firm risk. The coefficients on FSIZE are significantly negative and positive with Z-score and CFV, respectively. These results are consistent with previous studies that large firms take more risk due to the “too big to fail” problem [65, 66]. The value of these coefficients are quite high (-0.250, 0.150, and 0.001 in regressions 1, 2 and 3, respectively) indicating that large firms can take higher risk than small firms in MENA countries. Similarly, the coefficients on FAGE are positive and significant, with LEV and CFV indicating that a firm’s scope of operation increases firm risk. However, the effect of firm age on risk is not very significant because the value of coefficients of FAGE in regressions 2 and 3 is quite low. Finally, the coefficients of DEBT are significantly positive with LEV and CFV, respectively, indicating that debt structure (i.e., long-term debt) may increase firm risk. This finding does not support the previous studies that firms with more short-term debt usually take more risk [67, 68]. This is because managers in firms with more long-term debt do not worry much about problems in the far future and only focus on high-risk projects to increase the firm’s profits for immediate gains.

Furthermore, the coefficients of WGI are significantly negative and positive with Z-score and LEV, respectively, confirming that country governance quality can increase firm risk. The value of these coefficients are very high (-0.369 and 0.118 in regressions 1 and 2, respectively). This finding is generally in agreement with our contention that, under a high national governance system, corporate insiders are well protected, government extraction of private benefits is smaller, and firm managers tend to perform risk management efficiently [13, 14, 69, 70]. As a result, firms tend to borrow more to carry out potential projects without much concern about the high level of risk. Our empirical evidence thus supports the “twin agency model” presented by Stulz [70], emphasizing that the strength of the national governance system plays an important role in shaping a firm’s risk-taking strategies.

### 4.3 National governance quality and the relationship between COVID-19, firm risk and performance

To test hypotheses H3a and H3b, we estimated Eqs 3 and 4, respectively, using the FE estimation method, and the results are presented in Table 6. Regarding the impact of the COVID-19 crisis on firm performance, the results show that the coefficient on COVID is negative with ROA, ROE, and TBQ but only significant with ROA at the 10% level. Most importantly, the coefficient on COVID*WGI is positive and significant with ROA and TBQ, indicating that the negative effect of COVID-19 crisis on firm performance may become weaker with high national governance quality.

**Table 6:** Country governance quality and the impact of COVID-19 on firm risk and firm performance.

	ROA		ROE		TBQ		Z-score		LEV		CFV	
	(1)		(2)		(3)		(4)		(5)		(6)	
	Coef.	t-stat	Coef.	t-stat	Coef.	t-stat	Coef.	t-stat	Coef.	t-stat	Coef.	t-stat
COVID	-0.025**	-1.930	-0.318	-1.060	-0.384	-1.580	-0.610***	-5.320	0.022**	1.970	0.207***	2.370
WGI	0.101***	2.300	-0.575	-0.770	-0.510	-1.370	0.187	0.890	0.394***	3.450	0.063	0.090
COVID*WGI	0.056	1.760	0.800***	2.310	0.038**	2.110	0.072***	2.320	-0.185***	-2.340	-0.464**	-1.950
FAGE	0.001***	2.140	-0.004	-0.350	0.018**	2.050	0.001	0.290	-0.002**	-2.050	-0.019	-0.540
MTB	0.000*	-1.790	-0.006	-0.800	0.002	1.080	-0.000*	-1.850	0.000***	3.030	0.002	0.600
FSIZE	0.023**	2.090	0.223***	2.630	0.862***	2.680	0.178	1.010	0.041	0.880	1.519*	1.890
DIV	0.067***	5.890	0.019	0.080	0.110	0.620	0.159	1.280	-0.038	-1.220	-0.136	-0.130
TANG	0.000	0.820	0.001*	1.840	-1.824***	-5.050	0.000	-0.010	0.000	0.360	0.133***	3.080
DEBT	0.228**	2.070	2.456	1.020	3.118***	3.660	0.236	0.620	0.220***	4.140	-0.140	-0.140
LEV	-0.128	-3.120	-1.088	-1.090	-0.169	-0.090						
GDPC	0.033	1.540	-0.977***	-2.690	0.634	1.180	-0.083	-0.420	-0.312***	-5.540	-1.225	-0.810
Cons	-0.163***	-0.810	7.079	0.610	1.774	0.520	0.112	0.070	2.851***	5.210	2.150	0.210
Country fixed effect	yes		yes		yes		yes		yes		yes	
Industry fixed effect	yes		yes		yes		yes		yes		yes	
Obs	3253		3253		3253		3253		3253		3253	

Note: This table presents the results of the fixed effect estimates of Eq 3. Regressions 1–3 presents the Eq 3 estimation results that use firm performance variables (ROA, ROE, and TBQ, respectively) as dependent variables. Regressions 4–6 presents the Eq 3 estimation results that use firm risk variables (Z-score, LEV, and CFV, respectively) as dependent variables. See Table 1 for variable definitions. Superscripts *, **, *** indicate statistical significance at 10%, 5%, and 1% levels, respectively.

However, these results weakly support hypothesis H3a. Regarding the impact of the COVID-19 crisis on firm risk, we found that the coefficients on COVID are negative with Z-score but positive with LEV and CFV, and all coefficients are statistically significant. These results are consistent with those in Table 5.

Moreover, the coefficients on COVID*WGI are positive for Z-score and negative for LEV and CFV. These results indicate that countries with high governance quality can reduce the positive impact of COVID-19 on firm risk. The value of these variables is very high (0.072, -0.185 and– 0.464 in regressions 4, 5, and 6 respectively) indicating that national governance quality plays an important role in reducing firm risk. Overall, these results strongly support hypothesis H3b and are consistent with institutional quality theory and previous studies which indicate that country governance quality can increase firm management effectiveness [16, 17, 23]. Therefore, national governance quality can reduce firm risk through firm management effectiveness channel. In other words, the positive impact of the COVID-19 crisis on firm risk becomes weaker under high national governance quality. In addition, the coefficients of COVID in regressions 4–6 of Table 6 are consistent with the baseline results in Table 5, confirming that COVID-19 positively affects firm risk in general, providing further support for hypothesis H2. These findings contribute to the literature that country governance quality plays an important role in enhancing management effectiveness and reducing the adverse effects caused by economic shock, such as the COVID-19 crisis. As a result, country governance quality can become an environment for firms to operate sustainably. The coefficients on WGI are positive for ROA (regression 1) and positive for LEV (regression 5), while these coefficients are not statistically significant in regressions 2, 3, 4, and 6.

The unclear effect of the COVID-19 crisis on firm performance may be derived from the different effects of the crisis on firms in different industries. Thus, we separated our sample into two groups of industries. The first group (Group A) includes firms in industries whose operations during the pandemic period were supported by national governments or those that were less affected by the pandemic control policies. These industries include “Food,” “Health Care Equipment and Services,” “Pharmaceuticals, Biotechnology, and Life Sciences,” “Software and Services,” or “Telecommunication Services.” The second group (Group B) includes firms of other industries. Previous studies have found that these industries, which are called basic industry, are supported by the government to respond to the COVID-19 pandemic or may be less affected by lockdown regulations [71–74].

Table 7 reports the regression results of Eq 3 using the fixed-effect method. The coefficients of COVID are positive and significant with ROA and ROE in regressions 1 and 2, indicating that the COVID-19 crisis increased the performance of firms in Group A. On the other hand, the coefficients of COVID are negative and statistically significant with ROA, ROE, and TBQ in regressions 4, 5, and 6, indicating that the COVID-19 crisis reduced the performance of firms in Group B. Our results partially support hypothesis H1, that COVID-19 negatively affects the performance of most firms. Furthermore, the coefficients of COVID*WGI are positive with all regressions and statistically significant in regressions 2, 3, 4, and 6 in Table 7, indicating that national governance quality increases the positive impact of the COVID-19 crisis on the performance of firms in Group A and reduces the negative impact of this crisis on the performance of firms in Group B. These findings continue to support institutional quality theory and imply that national governance quality can reduce the effect of COVID-19 crisis on firm performance through management effectiveness channel. Overall, these results strongly support hypothesis H3a.

**Table 7:** COVID-19 crisis and firm performance by industry.

	Group A						Group B					
	ROA		ROE		TBQ		ROA		ROE		TBQ	
	(1)		(2)		(3)		(4)		(5)		(6)	
	Coef.	t-stat	Coef.	t-stat	Coef.	t-stat	Coef.	t-stat	Coef.	t-stat	Coef.	t-stat
COVID	0.042***	2.350	0.111**	2.070	7.457	1.019	-0.021***	2.290	-0.452*	-1.840	-2.339**	-1.980
WGI	0.070***	2.440	0.326	0.850	2.099***	2.380	0.076	4.000	-3.544	-1.280	2.100	0.700
COVID*WGI	0.005	0.400	0.057***	2.330	1.228***	2.320	0.005***	2.640	0.305	0.260	0.325***	2.250
FAGE	0.002	0.140	0.006	0.030	-0.853	-0.210	-0.007	-0.810	-0.062	-0.050	0.210	0.150
MTB	-0.000***	-3.790	-0.001	-1.040	0.001	0.090	-0.001***	-4.810	-0.031	-1.440	0.009	0.370
FSIZE	0.055*	1.860	0.379**	1.950	-8.672	-0.990	0.055***	2.990	3.466**	1.890	-2.881	-0.990
DIV	0.006	0.600	0.101	0.720	-6.552***	-2.130	0.018***	2.490	-0.072	-0.070	0.734	0.630
TANG	0.001	0.660	0.003	0.090	-3.182***	-5.070	-0.000	-0.360	-0.002	-0.130	-1.819***	-7.770
DEBT	0.011	0.700	-0.016	-0.070	3.300	0.690	-0.010	-0.990	0.398***	2.260	5.160***	3.120
LEV	-0.014	-0.780	0.094	0.380	4.010	0.740	-0.028***	-2.240	0.118	0.070	-2.306	-1.180
GDP	-0.035	-1.040	-0.016	-0.040	1.982	0.200	-0.021	-0.900	0.611	0.180	2.423	0.660
Cons	-0.145	-0.240	-3.209***	-2.400	86.089	0.490	-0.007	-0.020	-32.370	-0.570	-3.811	-0.060
Country fixed effect	yes		yes		yes		yes		yes		yes	
Industry fixed effect	yes		yes		yes		yes		yes		yes	
Obs	800		800		800		2453		2453		2453	

Note: This table presents the results of the fixed effect estimates of Eq 3. Regressions 1–3 present the estimation results for Group A, and regressions 4–6 presents the results for Group B. See Table 1 for variable definitions. Superscripts *, **, *** indicate statistical significance at 10%, 5%, and 1% levels, respectively.

Table 8 reports the estimation results of Eq 4. The results show that the coefficients of COVID are negative and statistically significant with Z-score (regression 4) and positive and statistically significant with both LEV and CFV in regressions 2, 3, and 6. These results imply that COVID-19 increases firm risk in most firms in MENA countries. In addition, the coefficients on COVID*WGI are positive with Z-score in regression 1 and negative with LEV and CFV in regressions 2, 3, 5, and 6, indicating that national governance quality can reduce the negative impact of the COVID-19 crisis on the risk of most firms in MENA countries.

**Table 8:** COVID-19 crisis and firm risk by industry.

	Group A						Group B					
	Z-score		LEV		CFV		Z-score		LEV		CFV	
	(1)		(2)		(3)		(4)		(5)		(6)	
	Coef.	t-stat	Coef.	t-stat	Coef.	t-stat	Coef.	t-stat	Coef.	t-stat	Coef.	t-stat
COVID	-0.458	-0.290	0.014**	2.051	0.001***	4.140	-0.908**	-1.950	0.152	1.130	0.003***	2.280
WGI	-0.121	-0.320	0.156***	2.450	0.002	0.460	-0.484**	-1.930	0.108***	3.050	-0.000	-0.940
COVID*WGI	0.133*	1.890	-0.103***	-3.610	-0.000***	-2.160	-0.116	-1.080	-0.022***	-2.450	-0.001**	-1.940
FAGE	0.207	1.120	0.004	0.120	0.002***	40.840	0.179	1.570	0.020	1.250	0.002	0.380
MTB	-0.000	-0.400	0.000**	1.960	-0.001	-0.110	0.001	0.390	0.000*	1.820	0.001	0.110
FSIZE	-0.403**	-1.930	0.011	0.170	-0.001	-0.810	-0.138***	-2.570	0.199***	5.880	0.001***	2.290
DIV	0.096	0.690	0.026	1.120	0.000	1.150	0.082*	1.830	-0.030***	-2.200	0.000	0.090
TANG	-0.031	-1.100	0.001	0.300	0.001	1.240	0.000	0.000	0.000	1.020	0.001	0.010
DEBT	-0.282	-1.370	0.235***	6.770	0.004	0.070	0.041	0.300	0.169***	8.850	0.000	0.070
GDPC	-0.338	-0.750	0.134	1.760	0.001***	6.110	-0.006	-0.020	0.039	0.900	-0.001***	-2.790
Cons	0.525	0.070	-1.330	-0.990	9.976***	14.000	-3.876	-0.750	-2.306***	-3.190	8.889***	15.000
Country fixed effect	yes		yes		yes		yes		yes		yes	
Industry fixed effect	yes		yes		yes		yes		yes		yes	
Obs	800		800		800		2453		2453		2453	

Note: This table presents the results of the fixed effect estimates of Eq 4. Regressions 1–3 present the estimation results for Group A, and regressions 4–6 present the results for Group B. See Table 1 for variable definitions. Superscripts *, **, *** indicate statistical significance at 10%, 5%, and 1% levels, respectively.

Overall, there is evidence that the COVID-19 crisis reduced the performance of firms in many industries and increased the firm risk in most industries. Furthermore, the country’s governance quality can prevent the effect of an economic shock like the COVID-19 crisis on firm operations in general. The findings provide important implications that regulators functioning in low country governance quality should have an appropriate policy to help firms reduce the impact of the COVID-19 crisis on firm operation as they may be affected harder than firms in countries with high institutional quality.

### 4.4 Robustness tests

#### 4.4.1 Alternative measure of Covid-19 and estimation method

In this study, we apply the natural logarithm of stringency index to measure the severity of COVID-19 crisis as a robustness test. Moreover, despite the consistently negative relationship between COVID-19 and firm performance and a negative relationship between COVID-19 and firm risk, our baseline results might suffer from endogeneity biases [75, 76]. First, to address the potential endogeneity problem, we applied the system GMM estimation method to Eqs 3 and 4. Based on the previous studies, we treat lag 1 year of firm risk and performance as well as the bank-specific controls as endogenous [16, 77]. For the variables that enter the dynamic panel models as predetermined their first and longer lags can be used as instruments [78]. To control the different impacts of the COVID-19 crisis on firms in different industries, we added the control variable INDS, which is 1 if firms are in a Group A industry and 0 otherwise, into Eqs 3 and 4. The results presented in Table 9 show that the COVID-19 crisis negatively impacted firm performance. The coefficients on SINDEX are negative and significant with firm performance variables in regressions 1 and 3, and positive and significant with firm risk variables in regressions 4–6. The signs of SINDEX coefficients are consistent with the results in Tables 7 and 8, further supporting hypothesis H2. Further, the coefficients on SINDEX*WGI continue to be positive with firm performance variables in regressions 1 and 2 and negative and significant with firm risk variables in regressions 5 and 6, which is clear evidence that country governance quality can reduce the positive impact of the COVID-19 crisis on firm risk as well as the negative impact of this crisis on firm performance. Although the coefficient of SINDEX*WGI is not statistically significant in regressions 3 and 4, it is correctly signed.

**Table 9:** Robustness test using system GMM results and alternative measure of COVID-19 crisis.

	ROA		ROE		TBQ		Z-score		LEV		CFV	
	(1)		(2)		(3)		(4)		(5)		(6)	
	Coef.	t-stat	Coef.	t-stat	Coef.	t-stat	Coef.	t-stat	Coef.	t-stat	Coef.	t-stat
SINDEX	-0.026**	-2.110	-0.119	-1.620	-0.347**	-1.960	-0.579***	-4.980	0.008***	2.420	-0.127***	-2.310
WGI	-0.071	-1.430	0.803*	1.860	-0.394	-1.020	0.058	0.310	0.229***	3.480	0.311	0.380
SINDEX*WGI	0.097***	2.470	0.719*	1.730	-0.657	-0.890	0.329	1.500	-0.031*	-1.710	-0.091**	-2.060
FAGE	0.001**	2.260	-0.005	-0.420	0.017*	1.710	0.002	0.500	-0.002**	-2.110	-0.017	-0.500
MTB	0.000**	-2.390	-0.005	-0.850	0.003	1.290	-0.000***	-2.460	0.000***	3.210	0.001	0.460
FSIZE	-0.019	-1.220	0.281*	1.730	-0.870**	-2.510	0.194**	2.130	0.106***	2.820	1.594	1.130
DIV	0.069***	7.210	0.007	0.030	0.125	0.630	0.160	1.200	-0.054*	-1.880	-0.114	-0.090
TANG	0.000	0.890	0.000	0.030	-1.835***	-5.560	0.000**	2.080	0.000	0.200	0.132***	2.410
DEBT	0.127***	2.570	0.925**	1.920	3.258***	2.860	0.228	0.620	0.223***	4.360	-0.178	-0.180
LEV	-0.105***	-4.280	-0.768	-0.850	-1.288	-0.610						
INDS	0.332**	2.120	0.331	0.810	3.471**	2.170	-1.281**	-2.240	-0.271	-1.130	0.520***	2.780
GDPC	0.036*	1.830	-1.132	-0.830	0.536	0.910	-0.091***	-2.450	-0.337***	-5.970	-1.363	-0.900
Cons	-0.198	-1.220	8.355	0.800	3.036	0.830	0.032	0.020	2.571***	5.390	2.895	0.310
Obs	3253		3253		3253		3253		3253		3253	
No of instruments	86		86		112		86		80		103	
AR2 (p-value)	0.612		0.474		0.337		0.133		0.860		0.376	
Hansen J (p-value)	0.167		0.918		0.140		0.124		0.121		0.814	

Note: This table presents the results of the system GMM estimates of Eqs 3 and 4. Regressions 1–3 present the Eq 3 estimation results that use firm performance variables (ROA, ROE, and TBQ, respectively) as dependent variables. Regressions 4–6 present the Eq 4 estimation results that use firm risk variables (Z-score, LEV, and CFV, respectively) as dependent variables. See Table 1 for variable definitions. Superscripts *, **, *** indicate statistical significance at 10%, 5%, and 1% levels, respectively.

Our system GMM estimator validity has been empirically checked using the Hansen-J over-identification test. The J statistic results reported in the last row of Table 7 across all models reject the null, confirming that the instruments (as a group) used in our system GMM model are valid. Moreover, the Arellano–Bond test, AR2 (p-values), shows no serial correlation in the second order. Overall, after addressing the potential endogeneity problem, the results are consistent with the baseline results, further supporting our hypotheses.

In addition, to investigate the heterogeneous impacts of the COVID-19 crisis on firm risk and performance, we applied the quantile regression method to Eqs 3 and 4 and the results are presented in Tables 10 and 11, respectively. Table 10 shows that the coefficients of SINDEX are negative and significant for ROA in regressions 1–3, with ROE in regressions 5, and 6, and with TBQ in regressions 9 and 10, while coefficients of SINDEX are not statistically significant in other regressions. These results indicate that the COVID-19 crisis had a negative impact on the performance of firms with low levels of performance. Furthermore, the coefficients of SINDEX*WGI are positive and statistically significant with ROA, ROE, and TBQ in regressions 1, 2, 5, 6, 9, and 10, indicating that country governance quality can help firms with low performance reduce the negative impact of the COVID-19 crisis on their performance. With low-performance firms, the results strongly support hypotheses H1 and H3a.

**Table 10:** Robustness test using the quantile regression method for firm performance.

	ROA				ROE				TBQ			
	Q25	Q50	Q75	Q90	Q25	Q50	Q75	Q90	Q25	Q50	Q75	Q90
	(1)	(2)	(3)	(4)	(5)	(6)	(7)	(8)	(9)	(10)	(11)	(12)
SINDEX	-0.001**	-0.002*	-0.008*	0.004	-0.004***	-0.008*	0.020	0.003	-0.007**	-0.036***	-0.032	-0.015
	(-2.30)	(-1.71)	(-1.87)	(0.47)	(-2.52)	(-1.87)	(1.50)	(0.18)	(-2.18)	(-2.47)	(-0.86)	(-0.20)
WGI	-0.004	0.005**	0.008**	0.008	0.001	0.009	0.015**	0.005	-0.013	0.025	0.003	-0.084
	(-1.50)	(1.98)	(2.32)	(1.14)	(0.18)	(2.44)	(2.24)	(0.43)	(-0.37)	(0.39)	(0.10)	(-1.33)
SINDEX*WGI	0.001**	0.002***	0.009	0.002	0.002**	0.006**	0.016	0.003	0.021***	0.043***	0.024	0.031
	(2.17)	(2.51)	(1.56)	(0.20)	(2.16)	(2.05)	(1.53)	(0.16)	(2.38)	(2.41)	(1.47)	(0.30)
FAGE	-0.000	0.000	0.000	0.000	-0.000	-0.000	0.000	0.001**	0.000	-0.001	-0.001	-0.000
	(-0.41)	(0.64)	(0.61)	(1.35)	(-0.71)	(-0.13)	(1.30)	(2.05)	(0.09)	(-0.46)	(-1.58)	(-0.21)
MTB	-0.002***	-0.001***	-0.000***	-0.000**	0.000	-0.000	-0.000	-0.000	0.001*	0.022***	0.062***	0.174***
	(-4.03)	(-5.87)	(-4.02)	(-2.36)	(0.56)	(-0.04)	(-0.51)	(-0.67)	(1.88)	(9.14)	(11.63)	(15.67)
FSIZE	0.011***	0.003	-0.009***	-0.010	0.019	0.011***	0.018***	0.017	-0.017	-0.004	-0.050*	-0.140**
	(3.80)	(1.07)	(-2.46)	(-1.47)	(2.72)	(3.00)	(2.80)	(1.38)	(-0.49)	(-0.06)	(-1.64)	(-2.26)
DIV	0.062***	0.055***	0.059***	0.057***	0.128	0.094***	0.077***	0.048***	0.292***	0.127	0.090**	0.118
	(6.42)	(6.95)	(2.22)	(6.02)	(3.72)	(9.25)	(8.75)	(2.90)	(6.35)	(1.49)	(2.15)	(1.39)
TANG	-0.000	0.000	0.000	-0.000	-0.000	0.000	-0.000	-0.000	-2.172***	-0.692***	-0.343***	0.033***
	(-0.11)	(0.94)	(0.20)	(-0.13)	(-0.28)	(0.54)	(-0.03)	(-0.23)	(-8.42)	(-7.76)	(-9.98)	(13.91)
DEBT	-0.016***	-0.019***	-0.039***	-0.051***	-0.012	-0.021***	-0.031**	-0.027	0.141**	0.043	-0.089	-0.109
	(-2.71)	(-3.88)	(-5.23)	(-3.49)	(-0.82)	(-2.72)	(-2.23)	(-1.06)	(1.98)	(0.33)	(-1.37)	(-0.83)
LEV	-0.049***	-0.052***	-0.054***	-0.083***	-0.082	-0.049***	-0.070***	-0.119***	0.065	0.070	-0.167*	-0.497***
	(-6.25)	(-7.78)	(-5.37)	(-4.27)	(-4.29)	(-4.87)	(-3.86)	(-3.53)	(0.69)	(0.39)	(-1.95)	(-2.85)
INDS	0.11***	0.23***	0.36**	0.43*	0.30**	0.32**	0.44*	0.44	1.23**	1.82*	2.15	4.13
	(2.23)	(2.19)	(1.92)	(1.81)	(2.16)	(1.93)	(1.89)	(1.61)	(2.11)	(1.93)	(1.55)	(0.78)
GDPC	-0.001	-0.003*	-0.005*	-0.011*	-0.005	-0.013***	-0.035***	-0.055***	0.019	-0.008	0.030	0.052
	(-0.42)	(-1.74)	(-1.85)	(-1.95)	(-0.94)	(-4.50)	(-6.76)	(-5.72)	(0.70)	(-0.16)	(1.24)	(1.05)
Cons	-0.093***	0.040*	0.203***	0.339***	-0.158	0.067**	0.310***	0.657***	1.254***	1.322**	1.617***	2.119***
	(-3.62)	(1.82)	(6.23)	(5.32)	(-2.52)	(2.04)	(5.23)	(5.95)	(4.04)	(2.29)	(5.75)	(3.72)
Obs	3253	3253	3253	3253	3253	3253	3253	3253	3253	3253	3253	3253

Note: This table presents the results of the quantile regression estimates of Eq 3. Regressions 1–4, 5–8, and 9–12 present the Eq 3 estimation results that use firm performance variables (ROA, ROE, and TBQ, respectively) as dependent variables. Q25, Q50, Q75, and Q90 are quantile 25^th^, 50^th^, 75^th^, and 90^th^, respectively. See Table 1 for variable definitions. Superscripts *, **, *** indicate statistical significance at 10%, 5%, and 1% levels, respectively.

**Table 11:** Robustness test using quantile regression method for firm risk.

	Z-score				LEV				CFV			
	Q25	Q50	Q75	Q90	Q25	Q50	Q75	Q90	Q25	Q50	Q75	Q90
	(1)	(2)	(3)	(4)	(5)	(6)	(7)	(8)	(9)	(10)	(11)	(12)
SINDEX	-0.229***	-0.177**	-0.129*	0.112	0.000**	0.002**	-0.009	-0.004	0.003	0.007***	**0.00388**	0.579**
	(-4.03)	(-2.19)	(-1.73)	(0.92)	(2.07)	(2.14)	(-0.67)	(-0.27)	(0.32)	(2.37)	(2.02)	(2.27)
WGI	-0.027	-0.012	0.068	0.036	0.000	0.031***	0.044***	0.012	-0.019***	-0.035**	-0.089	4.230**
	(-0.57)	(-0.17)	(1.08)	(0.36)	(0.01)	(2.95)	(3.89)	(1.11)	(-2.85)	(-2.15)	(-0.69)	(2.33)
SINDEX*WGI	0.153**	0.117**	0.000	0.066	-0.002**	-0.001**	-0.008	-0.008	-0.013**	-0.024**	-0.097***	-1.291***
	(1.95)	(2.06)	(1.61)	(0.40)	(-1.91)	(-2.03)	(-0.41)	(-0.47)	(-2.18)	(-1.99)	(-2.46)	(-2.43)
FAGE	0.004***	0.006***	0.005***	0.016***	0.000	0.001**	0.001*	0.000	0.001***	0.000	0.000	-0.040
	(2.66)	(2.83)	(2.93)	(5.50)	(0.01)	(2.36)	(1.65)	(0.73)	(4.46)	(-0.60)	(0.09)	(-0.77)
MTB	0.000	0.000	-0.000	-0.001	0.000***	0.001***	0.001***	0.001***	0.001***	0.001***	0.007***	0.000
	(0.44)	(0.03)	(-0.44)	(-0.37)	(2.82)	(4.12)	(4.20)	(2.96)	(9.42)	(3.41)	(3.01)	(0.01)
FSIZE	0.151***	0.272***	0.351***	0.708***	0.000	0.035***	0.011	-0.034***	-0.037***	-0.047***	0.059	3.512**
	(3.22)	(4.09)	(5.70)	(7.10)	(0.02)	(3.42)	(1.03)	(-3.18)	(-5.59)	(-2.92)	(0.47)	(1.97)
DIV	0.047	0.290***	0.355***	0.331**	-0.010	-0.082***	-0.062***	0.002	-0.076***	-0.077***	-0.111	-0.682
	(0.74)	(3.22)	(4.25)	(2.45)	(-1.35)	(-5.84)	(-4.10)	(0.12)	(-8.36)	(-3.55)	(-0.65)	(-0.28)
TANG	-0.001	-0.005*	-0.002	-0.003	0.000	-0.001***	0.000	0.000	0.305***	0.305***	0.344***	1.853***
	(-0.79)	(-1.87)	(-0.66)	(-0.71)	(0.14)	(-2.77)	(0.42)	(-0.18)	(9.92)	(4.91)	(7.10)	(7.16)
DEBT	0.128	0.119	0.415***	0.644***	0.289***	0.351***	0.188***	0.103***	-0.029**	-0.119***	-0.387	-0.936
	(1.35)	(0.88)	(3.32)	(3.18)	(6.83)	(6.77)	(8.37)	(4.71)	(-2.12)	(-3.65)	(-1.51)	(-0.26)
INDS	-0.54	-1.26**	-1.23***	-1.33**	0.09	1.16**	1.17***	1.25	0.47**	0.52*	-0.54	-1.26**
	(0.85)	(2.12)	(2.41)	(2.12)	(1.64)	(1.93)	(2.27)	(1.61)	(2.00)	(1.82)	(0.85)	(2.12)
GDPC	-0.035	0.017	-0.075	-0.122	0.000	-0.031***	-0.040***	-0.007	0.010*	-0.010	-0.117	2.017
	(-0.93)	(0.31)	(-1.53)	(-1.54)	(-0.02)	(-3.73)	(-4.54)	(-0.78)	(1.88)	(-0.79)	(-1.16)	(1.42)
Cons	-0.890**	-1.519**	-0.381	-2.227**	0.010	0.152	0.684***	0.929***	0.386***	0.907***	1.787	-37.455**
	(-2.08)	(-2.50)	(-0.68)	(-2.44)	(0.20)	(1.62)	(6.77)	(9.40)	(6.34)	(6.18)	(1.55)	(-2.30)
Obs	3253	3253	3253	3253	3253	3253	3253	3253	3253	3253	3253	3253

Note: This table presents the results of the quantile regression estimates of Eq 4. Regressions 1–4, 5–8, and 9–12 present the Eq 3 estimation results that use firm risk variables (ROA, ROE, and TBQ, respectively) as dependent variables. Q25, Q50, Q75, and Q90 are quantile 25^th^, 50^th^, 75^th^, and 90^th^, respectively. See Table 1 for variable definitions. Superscripts *, **, *** indicate statistical significance at 10%, 5%, and 1% levels, respectively.

Table 11 reports the quantile regression results for Eq 4 using firm risk, measured by Z-score, LEV, and CFV in most regressions as dependent variables. The coefficients on SINDEX are negative and statistically significant with firm risk variables. Specifically, the coefficients of SINDEX are negative with Z-score in regressions 1–3, positive with LEV in regressions 5 and 6, and positive with CFV in regressions 10–12. These results provide strong evidence that COVID-19 increases firm risk at all levels of risk and strongly supports hypothesis H2. Moreover, the coefficients of SINDEX*WGI are negative and statistically significant with firm risk in most regressions. Specifically, the coefficients of SINDEX*WGI are positive with Z-score in regressions 1 and 2, negative with LEV in regressions 5–6, and negative with CFV in regressions 9–12, indicating that country governance quality can reduce the impact of COVID-19 on firm risk. The sign of these coefficients is consistently negative through all quantiles, substantiating strong support for hypothesis H3b.

Regarding control variables, Tables 8 and 9 provide some important findings. In Table 8, the coefficients on DIV are positive with firm performance in most of the quantile of dependent variables. This finding is consistent with previous studies that find that dividends payouts are a good signal, implying that firms have good performance [79, 80]. Similarly, the coefficients on LEV are negative and significant with firm performance in most of the regressions, indicating that firm leverage is negatively associated with firm performance. Again, this finding is consistent with previous studies that find a negative relationship between leverage and profit performance [56, 81].

Furthermore, we find that dividend paid out is negatively associated with firm risk. Table 9 shows that the coefficients of DIV are positive and significant with Z-score in regressions 2, 3, and 4 but negative and significant with both LEV and CFV in regressions 6, 7, 9, and 10. Finally, the results in Table 8 and Table 9 show that the coefficients of WGI are significant with firm performance variables (Table 10) in regressions 2, 3, and 7 and significant with firm risk variables (Table 11) in regressions 6, 7, 9, 10 and 12. The role of country governance quality for business operations is to provide a good environment and reduce economic shocks.

#### 4.4.2 Alternative national governance quality measure

There are some existing arguments in the literature related to using overall country governance quality index. Langbein and Knack [82] point out that “the World Governance Index essentially measures the same underlying governance concept, although the six dimensions were meant to capture conceptually distinct dimensions.” As a robustness test, we followed the suggestion of Hou and Wang [43] and used each component of WGI, comprising “rule of law” (RL), “voice and accountability” (VA), “political stability and absence of violence” (PV), “government effectiveness” (GE), “regulatory quality” (RQ), and “control of corruption” (CC) and instead of the overall index, used one of the six country governance quality indexes. Specifically, we applied the system GMM method to estimate Eq 3 by using each of WGI components to measure country governance quality. In this test, we use ROA and Z-score to measure firm performance and risk, and the results are reported in Table 12. Because our previous findings are that the impact of the Covid-19 crisis on firm performance may be different between two groups of industry, we continue to add the INDS as control variables. After applying the alternative national governance quality measure, we find that the main results consistent with our baseline results. The diagnostics tests in Table 12 show that, as indicated by the Arellano–Bond test, AR2, and the Hansen J tests, all the regressions are valid.

**Table 12:** Robustness test results using system GMM and components of country governance quality.

	ROA						Z-score					
	RL	VA	PV	GE	RQ	CC	RL	VA	PV	GE	RQ	CC
	(1)	(2)	(3)	(4)	(5)	(6)	(7)	(8)	(9)	(10)	(11)	(12)
SINDEX	-0.008*	-0.012	-0.022**	-0.015	-0.007***	-0.006	-0.579**	-0.346***	-0.534**	-0.476**	-0.490*	-0.288
	(-1.88)	(-0.99)	(-2.18)	(-1.52)	(-2.65)	(-0.61)	(-2.36)	(-2.48)	(-2.18)	(-2.05)	(-1.80)	(-1.27)
WGI	-0.064**	-0.064**	-0.059*	-0.048*	-0.032	-0.086**	0.146	1.157***	-0.207	0.860**	2.263***	2.098***
	(-2.07)	(-2.22)	(-1.69)	(-1.78)	(-1.33)	(-2.10)	(0.59)	(5.56)	(-0.96)	(2.33)	(4.36)	(3.37)
SINDEX*WGI	0.012*	0.019*	0.016	0.012*	0.008**	0.014	0.139***	0.160*	0.016	0.026**	0.228**	0.229
	(1.65)	(1.73)	(1.58)	(1.76)	(1.95)	(1.60)	(2.68)	(1.73)	(0.11)	(2.14)	(1.90)	(1.21)
FAGE	0.000*	0.001*	0.001*	0.001**	0.001**	0.000	0.006	0.002	0.006	0.009	0.021***	0.005
	(1.67)	(1.74)	(1.68)	(1.96)	(2.12)	(1.32)	(1.13)	(0.31)	(1.23)	(1.55)	(2.89)	(0.91)
MTB	-0.000**	-0.000**	-0.000***	-0.000**	-0.000**	-0.000**	-0.000	-0.001*	0.000	0.000	0.001	-0.001
	(-2.11)	(-1.98)	(-2.61)	(-2.06)	(-2.06)	(-2.27)	(-0.22)	(-1.63)	(0.43)	(0.88)	(1.06)	(-1.44)
FSIZE	-0.012	-0.030*	-0.015	-0.017	-0.020	-0.017	-0.066	0.020	0.022	0.191	0.491*	-0.167
	(-0.63)	(-1.77)	(-0.81)	(-0.98)	(-1.32)	(-0.98)	(-0.31)	(0.13)	(0.12)	(0.89)	(1.79)	(-0.65)
DIV	0.060***	0.072	0.068***	0.062***	0.058***	0.067***	0.176	0.091	0.203	0.092	-0.030	0.137
	(6.00)	(6.52)	(6.53)	(6.66)	(6.52)	(6.43)	(1.18)	(0.68)	(1.38)	(0.63)	(-0.17)	(0.74)
TANG	0.000	0.000	0.000	0.000	0.000	0.000	0.000	0.000	0.000	-0.001	-0.001	0.000
	(0.53)	(0.82)	(0.92)	(0.55)	(0.50)	(0.67)	(-0.39)	(-0.02)	(-0.38)	(-0.62)	(-0.69)	(0.80)
DEBT	0.122**	0.211**	0.101**	0.082*	0.081*	0.153***	1.150**	0.089	1.167***	0.939**	1.048*	-0.105
	(2.31)	(2.41)	(2.06)	(1.73)	(1.68)	(2.71)	(2.36)	(0.33)	(2.81)	(2.04)	(1.82)	(-0.15)
INDS	0.251***	0.323***	0.345***	0.305***	0.351***	0.342***	-1.161***	-0.423	-1.264***	-1.232**	-2.341***	-0.389
	(4.31)	(4.23)	(4.31)	(4.32)	(3.26)	(4.32)	(-2.29)	(-0.77)	(-2.81)	(-2.31)	(-2.71)	(-1.40)
LEV	-0.103***	-0.124***	-0.092***	-0.091***	-0.090***	-0.107***						
	(-4.08)	(-3.57)	(-3.64)	(-4.04)	(-3.95)	(-4.06)						
GDPC	0.025	0.021	0.040**	0.022	0.028	0.046**	0.136	-0.177	0.146	0.041	0.534	-0.541
	(1.13)	(0.95)	(2.07)	(1.02)	(1.48)	(2.06)	(0.53)	(-0.84)	(0.54)	(0.18)	(1.38)	(-1.24)
Cons	-0.151	0.004	-0.254	-0.068	-0.104	-0.319	-0.235	2.377	-1.079	-1.407	-8.453**	7.359**
	(-0.97)	(0.03)	(-1.29)	(-0.51)	(-0.73)	(-1.53)	(-0.12)	(1.32)	(-0.44)	(-0.66)	(-2.54)	(2.00)
Obs	3253	3253	3253	3253	3253	3253	3253	3253	3253	3253	3253	3253
No of instruments	86	86	86	86	86	86	86	86	86	86	86	86
AR2 (p-value)	0.498	0.255	0.202	0.200	0.063	0.187	0.126	0.251	0.146	0.507	0.267	0.117
Hansen J (p-value)	0.139	0.115	0.213	0.128	0.187	0.109	0.115	0.213	0.165	0.211	0.143	0.125

Note: This table presents the results of the system GMM estimates of Eqs 3 and 4 using ROA (regressions 1–6) and Z-score (regressions 7–12) as dependent variables and using six components of national governance index. See Table 1 for variable definitions. Superscripts *, **, *** indicate statistical significance at 10%, 5%, and 1% levels, respectively.

The results in Table 12 show that the coefficients of SINDEX are negative and significant with ROA and Z-score in most regressions, while the coefficients of SINDEX*WGI are positive and significant in most regressions. Specifically, we found that the coefficients on the interaction variables of RL, VA, GE, and RQ are positive and significant in regressions 1, 2, 4, and 5, respectively, indicating that countries with higher quality of rule of law, voice and accountability, government effectiveness, and regulatory quality can reduce the negative effect of the pandemic on firm performance. Similarly, the coefficients on the interaction variables of RL, VA, GE, and RQ are positive and significant in regressions 7, 8, 10, and 11, indicating that countries with higher quality of rule of law, voice and accountability, government effectiveness, and regulatory quality can reduce the positive effect of the pandemic on firm risk. Our finding implies that countries in MENA region should focus on increasing the quality of rule of law, voice and accountability, government effectiveness, and regulatory quality to reduce the economic shock such as that caused by the pandemic, as better national governance quality can support firms during crises. Our research shows that not all elements of national institutions play the same role during covid-19. Specifically, there is no evidence that political stability and the absence of violence, and control of corruption can reduce the impact of the Covid-19 crisis on firm risk and performance. During covid-19, the issue of violence and political instability does not seem to be an issue facing companies in MENA countries. Furthermore, corruption can act as a "grease of wheels" in the economy, and make firms operate more efficiently [42]. Therefore, during the Covid-19 crisis, controlling corruption may make it more difficult for firms.

Overall, the results of the robustness tests support the notion that, even after using other proxies of country governance quality variables and controlling for unobserved heterogeneity, simultaneity, and dynamic endogeneity, we find strong evidence that the COVID-19 crisis negatively affected firm performance and positively affected firm risk. Further, country governance quality plays a moderating role in facilitating firm performance while reducing firm risk. Thus, the results are consistent with our expectations.

## 5. Conclusion

This study investigates the impact of the Covid-19 crisis on firm risk and performance and the role played by the country-level national governance system in facilitating the performance of firms by reducing their risks in MENA countries. After analyzing 739 non-financial listed firms in 11 MENA countries from 2011 to 2020, we found that the Covid-19 crisis has increased the firm risk in general and has negatively impacted the performance of low-performing firms. However, the national governance system has significantly reduced the impact of the Covid-19 crisis on the operations of firms. Our findings are consistent with the notion that national governance quality provides a good environment for firms to operate in and reduce the effects of economic shock on them.

In addition, our findings supply important implications for the shareholders of firms as well as the country’s regulators. First, shareholders and managers of firms with low performance should take necessary actions to improve their operational efficiency amid the complicated Covid-19 situation as low-performing companies are the ones affected most by the crisis. Second, firms need to focus on enhancing their risk management effectiveness to reduce the impact of the Covid-19 crisis on firm risk in general. Finally, the regulators in different countries need to design and implement long-term plans to enhance their governance quality as that plays a significant role in reducing the adverse effects caused by economic shocks. Furthermore, regulators in countries with low levels of governance quality should prioritize policies to support the firms’ activities, thereby avoiding systematic collapse. Moreover, these countries should focus on increasing the quality of rule of law, voice and accountability, government effectiveness, and regulatory quality to reduce similar economic shocks in the future.

Due to unavailable data of unlisted firms in MENA countries, some countries in our data have a small sample. Therefore, our study may not be suitable to investigate the impact of COVID-19 crisis on risk and performance of small- and medium-sized firms with in these countries. In addition, there are better ways to measure the impact of COVID-19, such as the number of days of lockdown applied in MENA countries and the level of pandemic control applied by countries, which are not available yet. Future research can extend our study by investigating the impact of COVID-19 on SME operations and examining the impact of COVID-19 from different perspectives.

## Supporting information

S1 File (XLSX)

S1 Appendix (DOCX)
