# Supplementary material for: The impact of COVID-19 on firm risk and performance in MENA countries: Does national governance quality matter?
Source: PLoS One. 2023 Feb 6;18(2):e0281148. doi: 10.1371/journal.pone.0281148 (PMC9901813; doi:10.1371/journal.pone.0281148)
Supplement: S1 Appendix — (DOCX) [file pone.0281148.s002.docx]

**Appendix 1**: Sample distribution

| Country | No of firms | % | Observations | % |
| --- | --- | --- | --- | --- |
| Bahrain | 16 | 2.17% | 66 | 2.03% |
| Egypt | 106 | 14.34% | 408 | 12.54% |
| Jordan | 64 | 8.66% | 251 | 7.72% |
| Kuwait | 50 | 6.77% | 215 | 6.61% |
| Morocco | 32 | 4.33% | 157 | 4.83% |
| Oman | 38 | 5.14% | 141 | 4.33% |
| Qatar | 16 | 2.17% | 74 | 2.27% |
| Saudi Arabia | 122 | 16.51% | 664 | 20.41% |
| Tunisia | 43 | 5.82% | 224 | 6.89% |
| Turkey | 211 | 28.55% | 883 | 27.14% |
| United Arab Emirates | 41 | 5.55% | 170 | 5.23% |
| Total | 739 | 100.00% | 3253 | 100.00% |

**Appendix 2:** List of industry

| No | Industry |
| --- | --- |
| 1 | Automobiles & Components |
| 2 | Capital Goods |
| 3 | Commercial & Professional Services |
| 4 | Consumer Durables & Apparel |
| 5 | Consumer Services |
| 6 | Food & Staples Retailing |
| 7 | Food, Beverage & Tobacco |
| 8 | Health Care Equipment & Services |
| 9 | Household & Personal Products |
| 10 | Materials |
| 11 | Media & Entertainment |
| 12 | Pharmaceuticals, Biotechnology & Life Sciences |
| 13 | Retailing |
| 14 | Software & Services |
| 15 | Technology Hardware & Equipment |
| 16 | Telecommunication Services |
| 17 | Transportation |
